# To the Editors of the Medical and Physical Journal

**Published:** 1804-04-01

**Authors:** Edward Hardman

**Affiliations:** Manchester


### *To the Editors of the Medical and Physical Journal.*

GENTLEMEN,

THE several methods employed by surgeons for the opening of an abscess are three: 1. Seton. 2. Caustic. 3. Incision; each of which has its advantages and disadvantages, well-known to every practitioner in surgery, but unnecessary here to be enumerated. I mean to add to these a *fourth*, which, as far as I know, has not yet been brought into general use. This is no other than the common cupping-glass, exhausted of air in the usual manner, and applied over the abscess, at the time thought proper for its operation. By this contrivance the whole contents of an abscess, however considerable, may be very speedily evacuated, and the first discharge is made with a very considerable force, similar to an explosion. Of the safety of the practice no one will I think doubt; and of its utility I can speak from experience. But, it may be asked, why multiply means when those already known are amply sufficient? To this question I should reply, that, as the opening of an abscess by the cupping-glass is momentary, it is much less painful than the caustic; and, in timid persons, we are sometimes prevented from adopting either the seton or incision, from their dread of every thing that relates to the knife.

The selection of proper cases for the application of it I leave to the judgment of the surgeon himself, only further suggesting, that, besides the superseding of the lancet in many instances, it may be had recourse to advantageously for extracting the slough, or deposition of coagulable lymph, in the furunculus or bile, by the detension of which the pain of that disease is protracted, and the cure often long retarded. I once knew a country bone-setter who was famous for sucking tumours of this description; but it is obvious that he would have lost all his business in that line had this method occurred to the practitioners in his neighbourhood, as it would have been quite as effectual as his in extracting the core, without giving any offence to delicacy. To save the patient however from unnecessary pain, it may be proper to add one caution, which is, not to exhaust the glass of air more than is necessary to rupture the integuments; and as every square inch of an exhausted receiver is deprived of an external compressing force, equivalent to almost fifteen pounds avoirdupois, a proportionable degree of force may be calculated to exert itself from within, in bursting the external covering of the abscess. And any force beyond this would not only aggravate the pain of the operation, but might occasion a greater flux of blood from the ruptured vessels of the part, than, under circumstances of extreme debility, it might be prudent to produce.

From analogy too I have also used the cupping-glass after the application of leeches; and where it is desirable to make the discharge by them suddenly, or in a greater quantity than from the paucity of leeches could be expected to flow, it will be found a valuable expedient.

*Manchester, Jan.* 6, 1804. EDWARD HARDMAN.

---

